# Cranial reconstruction in the literature: A CiteSpace visualized bibliometric analysis

**DOI:** 10.1007/s10143-025-03878-3

**Published:** 2025-10-11

**Authors:** Jake Barsch, Erion Sulaj, John L. Kilgallon, Robert Kamil, Nitesh V. Patel, Ira M. Goldstein

**Affiliations:** 1 Department of Neurosurgery, Hackensack Meridian School of Medicine, 123 Metro Blvd, Nutley, NJ 07110 USA; 2 Department of Neurosurgery, University of Miami Health, Miami, FL USA; 3 Department of Neurosurgery, HMH-Jersey Shore University Medical Center, Neptune, NJ USA; 4 Department of Neurosurgery, Hackensack University Medical Center, Hackensack, NJ USA

**Keywords:** Cranioplasty, Bibliometrics, CiteSpace, Cocitation, Alloplastic material, Autologous bone

## Abstract

Cranioplasty (CP) research has shown that the material used for its bone flap formation is not standardized, with several viable options. The aim of this study was to explore the development of research in cranioplasty from a bibliometric perspective, with a focus on material choice, to elucidate trends in CP literature over time. Original studies and review articles on cranioplasty material related research were obtained from the Scopus database from 2014 to 2024. R package “bibliometrix” was used to summarize main findings, examine the occurrence of top keywords, and visualize collaboration networks between countries. VOSviewer software was applied to conduct both co-authorship and co-occurrence analyses, with CiteSpace used to identify the references and keywords with the strongest citation bursts. A total of 933 publications on cranioplasty material were included. An analysis of publication titles demonstrated an increase in CP for traumatic brain injury (TBI) post-2010 along with the growth of custom implants (i.e., “patient-specific”). Early citation bursts focused on the application of various materials, while later keywords concerned surgical complications and clinical outcomes. Several authors had significant bursts of contribution, and the United States was the largest contributor to the existing body of literature. This bibliometric study elucidated keyword trends as they relate to CP and material selection. It mapped a fundamental knowledge structure consisting of countries, institutions, authors, journals, and articles in the research field of cranioplasty material over the past ten years. The results provide a comprehensive perspective about the wider landscape of this research area.

**Supplementary Information:**

The online version contains supplementary material available at 10.1007/s10143-025-03878-3.

## Introduction

Cranioplasty is a procedure that is designed to restore the natural shape and protective functions of the skull after a trauma or surgery that requires skull removal. Its use is important to allow normal restoration for the patient’s head shape while also providing a protective barrier for the brain [1]. Such repair can allow for structural support, restore the normal cerebrospinal fluid dynamics, and prevent dysregulation of blood flow [1–4]. A key factor in cranioplasty is selecting the right material, which must be durable, lightweight, flexible, easy to attach to the skull, and capable of supporting bone growth.

There are several options that can be used to perform cranioplasties including autologous bone and a variety of alloplastic materials, which are often chosen based on etiology, locations, and common complications. Autologous bone has the lowest rate of rejection and high viability, but is less effective for complex shapes and large defects [1, 5, 6]. The common alloplastic materials include titanium mesh, polyetheretherketone, hydroxyapatite, methyl methacrylate, and calcium phosphate bone cement, each with their own distinct advantages and drawbacks [1, 7–11]. Ultimately, it is crucial to choose a material that decreases potential complications while providing the greatest possible benefits at the best cost [1, 7, 8]. 

One way to analyze the usage and popularity of these different materials in cranioplasty is through a bibliometric analysis. Such studies employ both mathematical and statistical methods in scholarly journals to examine citation relationships between articles, high impact factors for a certain number of years, and the main topic clusters. Specifically, CiteSpace acts as a data visualization tool to conceptualize knowledge domains as mapping functions, identifying the highly cited and pivotal points [12]. These functions are more than simple counting, a common misconception of bibliometrics. Instead, they provide in-depth of information on the evolving focus of CP literature [13]. 

This manuscript aims to apply bibliometric analysis to the existing literature regarding cranioplasty materials, focusing on the last 10 years of research, to elucidate important data such as evolving collaboration networks, the reach of previous literature, and key contributors by country and journal. Such trends in literature can gauge the current state of the field and allow for meaningful analysis, identification of existing research gaps, and offer future direction of study.

The overall goal of this study is to examine these trends in order to inform future research endeavors, and is not intended to provide surgical decision-making guidance. Rather, the value of this bibliometric analysis lies in its ability to evaluate changes in CP literature over time to reveal overlapping and developing approaches to CP discourse, identifying both the evolution of subjects of investigation as well as the changing geography of such endeavors.

## Methods

### Data source

Scopus was used for data retrieval, as our data analysis tool only accepts input from a single database. Scopus was chosen due to its wider coverage of academic literature than the Web of Science and its recognition as the largest abstract and citation database in the world [14]. Therefore, it likely provides broader coverage of relevant literature than other database options.

All data was retrieved August 14, 2024 from the Scopus database. The literature search parameters were set with a focus on related keywords within the title, abstract, or indexed keywords of each article. Each was required to contain one or more of cranioplasty, craniofacial reconstruction, cranial reconstruction, skull reconstruction, or calvarial reconstruction and one of alloplastic material, titanium, titanium mesh, polymethyl methacrylate, porous polyethylene, bone cement, autologous bone, vascularized bone, non-vascularized bone, avascular bone, bone graft, polyetherketone, peek, or mma. These words encompassed all of the main materials used in CP and with its common accepted synonyms. A time frame of 2014–2024 was chosen to focus on recent data for an accurate understanding of the current state of the field. However, the citation data mapped by CiteSpace does not restrict to this time frame. This allows us to understand the history of CP research in the context of recent publications.

**Fig. 1:**
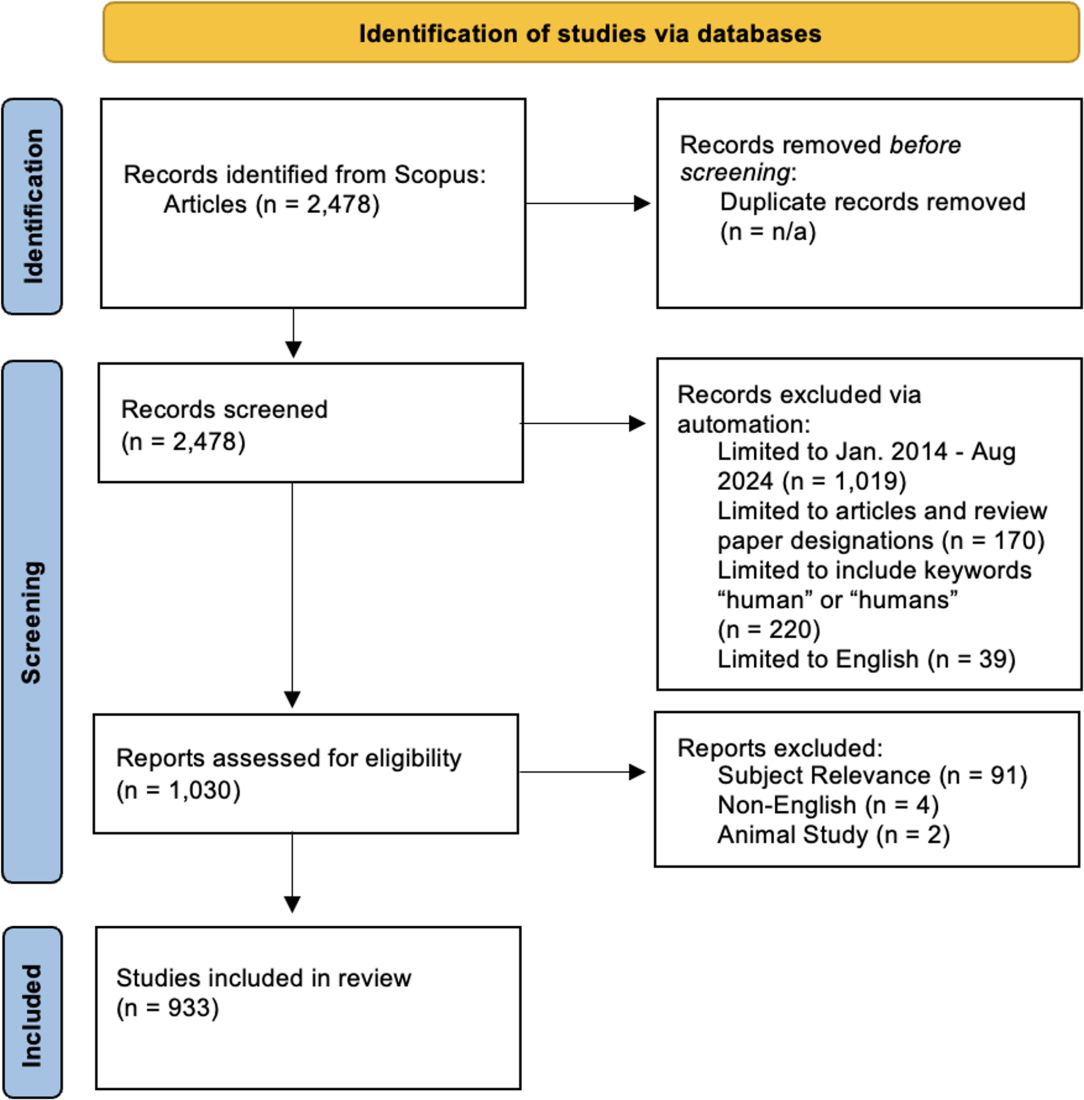
PRISMA Diagram

Our initial search yielded 2,478 results (Fig. 1). We then filtered to our chosen time frame of 2014–2024, which reduced the results to 1,459. We included both articles and review papers to better trace co-citations and sources, as review articles are often cited in lieu of their original study counterparts. This limited our results to 1,278. We then filtered for the keywords “human” or “humans” to limit animal study results, as well as English language publications, which left 1,030 articles. The exact search query is located in supplemental materials.

We then examined each result to ensure it met our inclusion criteria. This criteria required all of the following: (1) the article is a human study/review, (2) the article is in English, (3) the article involves CP, and (4) the article implicates one or more CP materials in its analysis. This examination eliminated 97 articles, and 933 were imported into CiteSpace.

### Data analysis

CiteSpace (6.3.R3) was used to convert the format of the included documents to a readable.txt, and calculate centrality of authors, keywords, countries, and co-cited references. This information was then visualized in chronological maps to show clustering, connections, and reveal “bursts” in certain keywords and cited references. The years per slice function was set to 1 year, with a K value of 25 for statistical analysis by the program. Different data groupings, mentioned above, were selected to reveal desired map information. Frequency scores were restricted to 40 + for keywords and 90 + for co cited authors. Clusters were created using the log-likelihood ratio (LLR) method. Node size was proportional strength of correlation.

## Results

### Keyword analysis

**Fig. 2:**
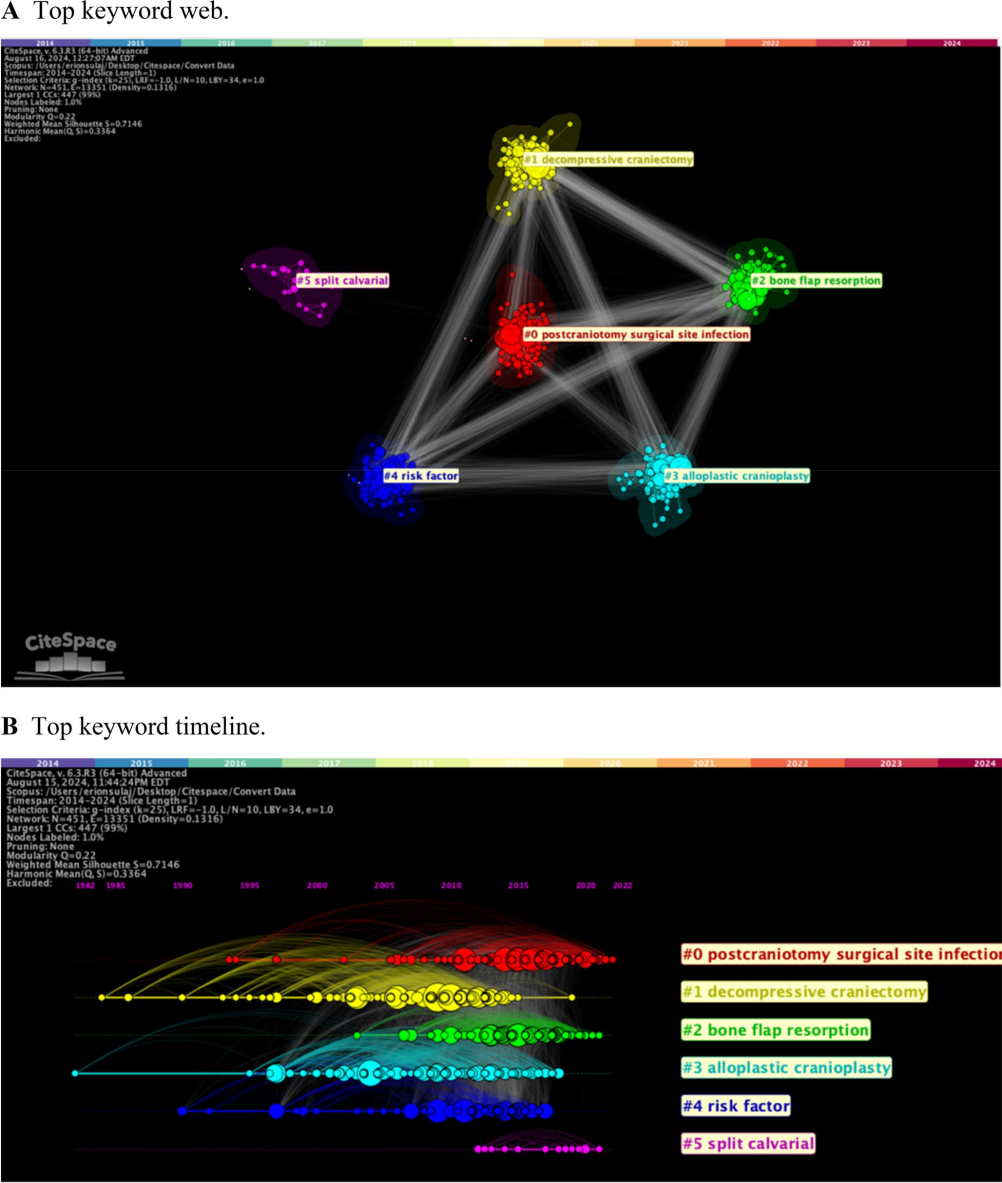

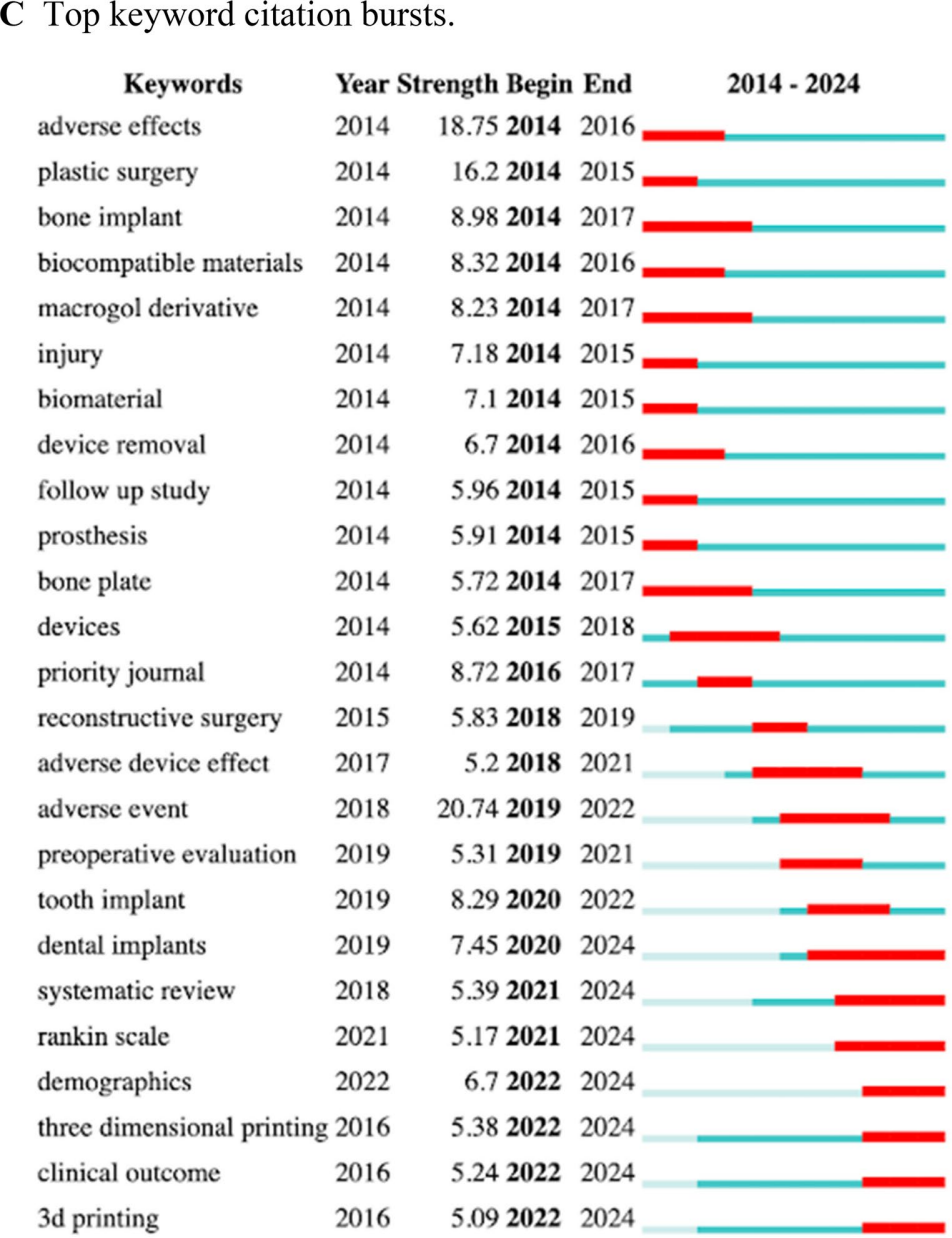
Top keyword characteristics **A**. Top keyword web **B**. Top keyword timeline **C**. Top keyword citation bursts

Keyword clusters in CP articles from 2014 to 2024 were mapped (Fig. 2A). The keyword “split calvarial” was often used in earlier studies concerning CP and materials in the mid 2010’s, but ideas such as “risk factors” and “site infection” came to dominate CP discourse. This coincided with the growth of alloplastic materials used in CP, possibly due to the growth prevalence in bone flap resorption research. The strength of cluster links, indicated by the white lines between each, reveal that these keywords are heavily linked, suggesting a central focus of modern CP investigations. The recurrence of top keywords over time through co-citation analysis in a timeline view was also created (Fig. 2B). Each horizontal line is connected to one of the top keywords, and node size illustrates the amount of publications at a given time that use the keyword. The overarching lines represent “links” from more recent publications back to previously cited papers that also contain the keyword. This creates a map that reveals the interconnected network of co-citations within and between each top keyword. This method affords a unique perspective on how CP focus has developed over time. “Split calvarial” which clusters in 2016 reappears in 2020. However, its prevalence pales next to the rest of the top keywords. While “decompressive craniectomy” and “alloplastic cranioplasty” do not cluster until post-2010, they have been a focus of research as far back as the 1980’s. On the other hand, questions on “risk factor” and “bone resorption” do not prevail until about 2015, where they dominate. There is a clear increase in CP for traumatic brain injury (TBI) post-2010 along with the growth of custom implants (i.e., “patient-specific”). At the same time, CP discourse for decompressive craniectomy seems to halt, suggesting general consensus regarding this procedure.

Citation bursts of keywords were also assessed (Fig. 2C). These bursts are timeframes where the keyword was used significantly more than average in our data set. Burst strength correlates to the relative quantity of a specific increase in publications for a given keyword. A higher score indicates that there was a more significant peak compared to other bursts in the dataset. Keywords involving autologous bone burst earlier in our search query than those regarding 3D printing and clinical outcome analysis, which shows the effect of growing technology and increased focus patient-outcomes. The top 17 relevant keywords in the data set were determined (Table 1); “bone graft” and “titanium/titanium mesh” were the most common material keywords used, likely pointing to a greater prevalence in their clinical use. The frequency of CP material-related keywords were mapped as well (Supplementary Fig. 1). The circular nature of the visualization implies that much of the research regarding CP in 2014 is still relevant to today’s studies. The quantity of lines also reveals a strong citation-linkage of material-related keywords, alerting to heavy reliance on foundational CP work to inform current understandings.

**Table 1:** Top relevant keyword table

Rank	Keyword	Citations	Centrality
1	cranioplasty	640	0.05
2	bone graft	352	0.03
3	Computer Assisted Tomography	281	0.03
4	Titanium	273	0.03
5	Craniotomy	263	0.02
6	Prostheses and Implants	170	0.01
7	Surgical Flaps	160	0.01
8	Prostheses and Orthoses	153	0.01
9	poly(methyl methacrylate)	137	0.01
10	Polyetheretherketone	117	0.01
11	Bone Transplantation	110	0.01
12	Hydroxyapatite	108	0.01
13	Surgical Mesh	88	0.01
14	Polymethyl Methacrylate	79	0.01
15	Bone cement	73	0.01
16	Polyethylene Glycols	47	0
17	Titanium Mesh	42	0

*Removed common / non-relevant words

## Citation and publication analysis

**Fig. 3:**
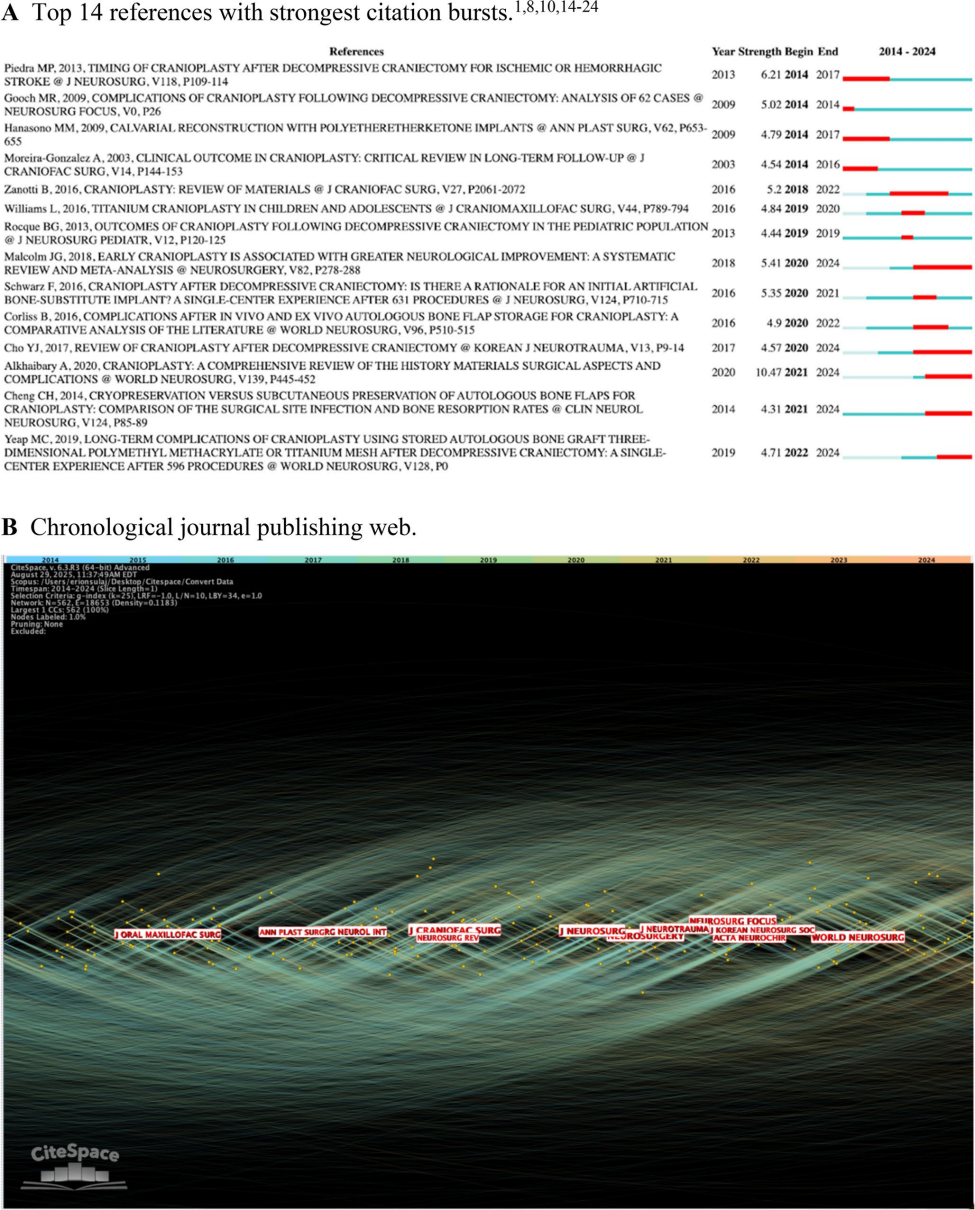
Top citation and publication characteristics **A**. Top 14 references with strongest citation bursts **B**. Chronological journal publishing web

The ten most co-cited articles were determined (Table 2) [15–24]. Six of ten are from the Journal of Neurosurgery, suggestive of the consequence of the greater impact factor of the journal. The majority of the top cited articles focus on clinical outcomes/complications, which aligns with the greater focus on such aspects in the last few years. Citation bursts of articles involving CP materials were also assessed (Fig. 3A) [1, 18, 20, 24–34]. In the early portion of our time frame, articles focused on CP methodology were the most popular. However, this shifted towards material review and analysis of CP complications closer to the present day. This indicates a development of interest away from the procedural aspects of CP, suggestive of a consensus on CP techniques, and towards a focus on patient outcomes and cost-effectiveness data.

**Table 2:** Most Co-cited articles [5–13]

Rank	Article	Citations	Centrality	Source Journal
1	Gooch et al., 2009^8^	77	0.08	Journal of Neurosurgery
2	Chang et al., 2010^7^	68	0.07	Journal of Neurosurgery
3	Grant et al., 2004^9^	61	0.04	Journal of Neurosurgery
4	Aydin et al., 2011^5^	53	0.09	Journal of Neurosciences in Rural Practice
5	Cabraja et al., 2009^6^	52	0.06	Journal of Neurosurgery
6	Zanotti et al., 2016^14^	52	0.03	Journal of Craniofacial Surgery
7	Yadla et al., 2011^12^	50	0.04	Neurosurgery Journal
8	Shah et al., 2014^11^	49	0.05	Journal of Neurosurgery
9	Zanaty et al., 2015^13^	49	0.03	Journal of Neurosurgery
10	Moreira-Gonzales et al., 2003^10^	48	0.08	Journal of Craniofacial Surgery

The contributions of different journals to CP material literature over time were depicted (Fig. 3B). The circular nature of the visualization means that journals are responsible for several publications among our data set. Details of the top journals and their citation frequencies were determined as well (Table 3). Neurosurgery and the Journal of Neurosurgery were the most cited journals along with the Journal of Craniofacial Surgery. All had centrality scores > 0.08, indicating a greater value of importance, or node size, than the other highly cited journals of the top 10.

**Table 3:** Journal citation frequency and centrality

Rank	Title	Citations	Centrality
1	Neurosurgery	466	0.18
2	Journal of Neurosurgery	457	0.09
3	Journal of Craniofacial Surgery	399	0.1
4	World Neurosurgery	358	0.08
5	Neurosurgery Focus	294	0.08
6	Plastic Reconstructive Surgery	294	0.05
7	Acta Neurochirurgica (Wien)	249	0.05
8	British Journal Neurosurgery	248	0.06
9	Journal of Clinical Neuroscience	241	0.04
10	Journal of Craniomaxillofacial Surgery	192	0.03

## Contribution analysis

**Fig. 4:**
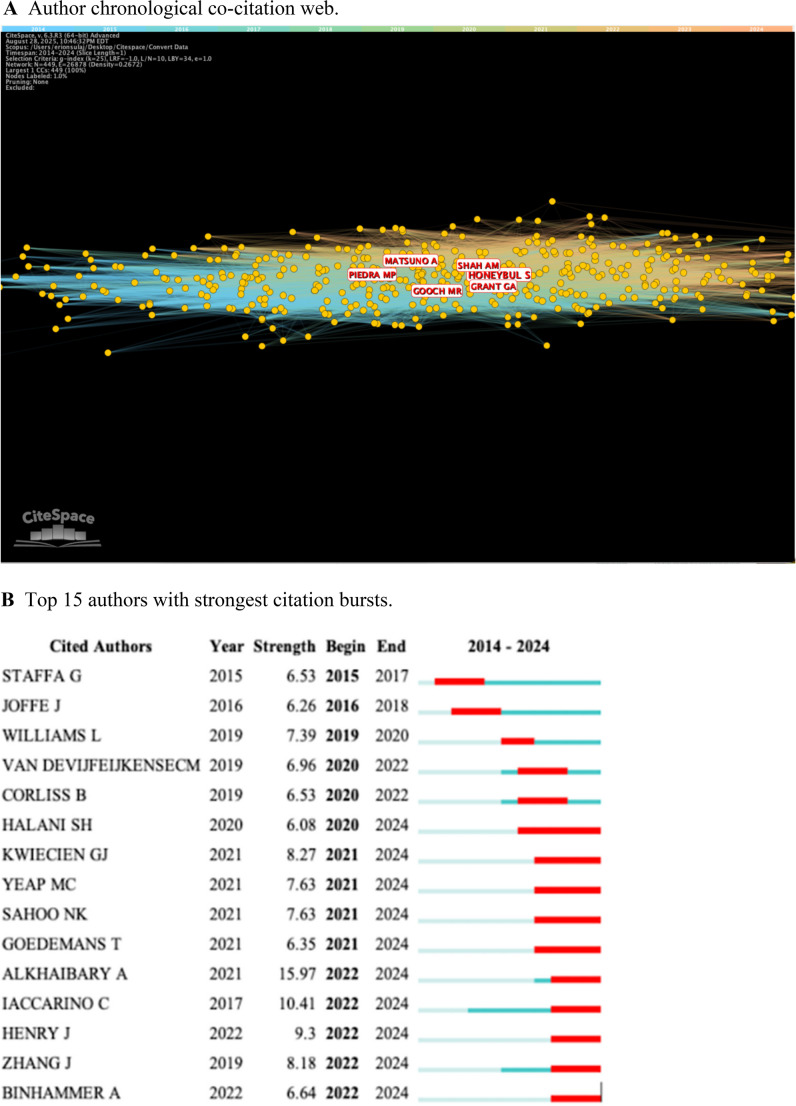

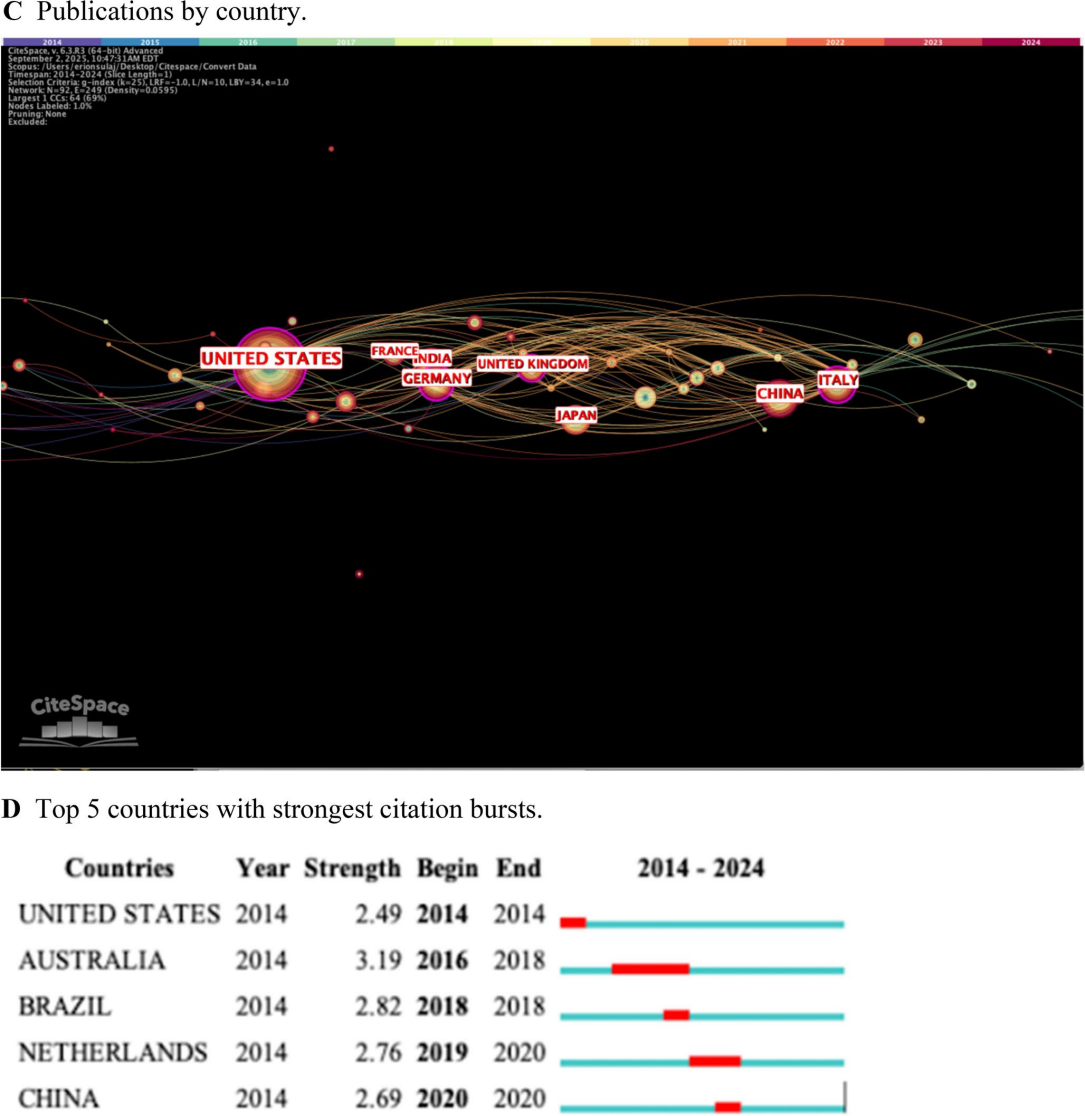
Authorship characteristics **A**. Author chronological co-citation web **B**. Top 15 authors with strongest citation bursts **C**. Publications by country **D**. Top 5 countries with strongest citation bursts

The contributions of different authors from 2014 to 2024 was determined (Fig. 4A). Node connections equate to the amount of co-citations received by different authors. Honeybul, S. appeared to make the most meaningful contribution to CP literature with a co-citation score of 209 (Table 4). However, no author had a centrality score greater than 0.06, indicating a low level of direct collaboration. Citation bursts by the author may be used to assess impact over time (Fig. 4B). Authors such as Staffa, G. and Joffe, J. had early citation bursts in our time frame with moderate strength (> 6.0). However, later contributors such as Alkhaibary, A. and Iaccarino, C. had extremely strong citation bursts (> 10.0) over the last two years, indicating highly relevant conclusions in their respective works.

**Table 4:** Individual author citations and centrality

Rank	Author	Citations	Centrality
1	Honeybul S	209	0.06
2	Gooch MR	120	0.03
3	Grant GA	118	0.03
4	Matsuno A	108	0.04
5	Shah AM	104	0.04
6	Malcolm JG	101	0.03
7	Schuss P	98	0.03
8	Lethaus B	97	0.04
9	Chang B	96	0.03
10	Yadla S	96	0.03
11	Zanotti B	96	0.02
12	Aydin S	92	0.03
13	Piedra MP	90	0.03

CP material literature was also mapped by country of authorship (Fig. 4C). Since 2014, the United States has contributed the greatest amount of papers with over 250 individual publications. China, Italy, Germany, and India make the subsequent greatest contributions. The United States overall centrality score is 0.35, revealing a stronger level of direct collaboration within the country. These scores are relative only to the country analysis, so centrality score stratification is what implies level significance. Therefore, Italy and Germany have centrality scores > 0.2, indicating significant intra-national collaboration. The low centrality scores of China and India (< 0.1) suggests non-significant collaboration with other nations. Citation burst by country of authorship was examined (Fig. 4D). Australia had the strongest burst between 2016 and 2018, indicating a geographical peak in CP research. However, each of the top 5 bursts have strengths of < 3.50 implying a lack of a true peak in CP interest in a country dependent manner.

## Discussion

Literature discourse regarding CP has significantly evolved over the last decade, and the visualization of these changes using the CiteSpace mapping software provides direct insights toward those changes. Keywords in the early 2010’s focused on decompressive craniectomy in relation to CP application, and its abrupt decrease in occurrence indicates changing investigation of CP and its operative potential with new ideas focused on patient specific implants and their complication rates. The keyword burst analysis of our dataset supports this notion, with “clinical outcome” and “adverse event” having particularly strong citation bursts at the turn of the decade. The top cited clinical keywords regarding CP also reflect the growing focus on alloplastic material use, as their citation prevalence has increased over time. This evolution underscores that bibliometric trends are not only academic, and that they can mirror real-world clinical shifts toward evaluating how material choice impacts complication rates, functional recovery, and ultimately long-term patient quality of life.

The type of material used in CP is not uniform, with many relevant and clinically valid options. Our analysis showed that autologous bone was the most frequent material mentioned, with titanium mesh in a close second. The alloplastic material options such as methyl methacrylate, hydroxyapatite, and polyetherketone occurred in article keywords with nearly 50% less frequency. However, added together, these options had over 352 keyword hits, which is greater than the sum for “bone graft.” This means that, as a whole, alloplastic materials are represented in the literature as often as that of autologous bone, but the type of alloplastic material used is non-standardized. Similarly, alloplastic materials were cited with greater prevalence over time, linking to their growth in popularity. This bibliometric evidence can be tied to surgical decision making, as surgeons are increasingly tasked with weighing long-term risks and costs when selecting the optimal material for a given patient [15]. 

The mapping of co-citations is one of CiteSpace’s strongest features, unearthing literature overlap and high impact studies. When examining a burst analysis, citation frequency in the early portion of the timeframe focused on methodology and application of CP. However, later bursts investigated clinical outcomes and complications. This trend implies a shift in academic interest from procedure development toward optimal patient outcomes. Recent studies have provided important information toward this new goal. For example, Belzeberg et al. analyzed 500 consecutive cranioplasty cases and demonstrated that infection, epidural hematomas, wound dehiscence, and seizures as the most common complications [35]. Similarly, Singh et al. examined cranioplasty outcomes following decompressive craniectomy and reported subgaleal collection, hydrocephalus, seizure, bone flap infection, intracerebral hematoma, empyema, and subdural hematoma as the most common complications [36]. Finding and understanding complications is integral to improving patient outcomes, as it can help refine surgical techniques to mitigate complications and for guide material selection for better long term results.

Citespace also highlights the contributions of individual articles within the CP literature. Regarding these articles, no study had a significant centrality score, meaning no single article made a notable contribution over the others. This is likely due to the breadth of CP and its various applications. Because there are many valid reasons for implementing CP, no singular article is wide enough in its scope to be universally applicable. Such a lack of central dominance suggests that decision making in clinical practice remains highly individualized, reinforcing the need for a standardized framework that synthesizes findings into actionable guidance for surgeons.

Data including author, country, and journal information was another valuable tool to understanding the development of CP research. Several authors made strong contributions to the field of CP research, with six authors amassing over 100 citations in their respective CP studies. In terms of author-based citation frequency, Alkhaibary, A. and Iaccarino, C. had extremely strong bursts from 2022 to 2024 [1, 37]. Alkhaibary, A. et al. published an in-depth CP review article including both material choice and clinical outcomes in 2020, which made a significant impact by summarizing the current state of the field [1]. As of our retrieval date, it has been cited over 150 times in just four years. Similarly, Iaccarino, C. et al. published an article in 2020 that examined CP post decompressive craniectomy, which has been cited over 80 times [15]. These high impact studies have heavily influenced the newest wave of CP research. In terms of contribution by country, the United States published nearly triple the amount of studies as China, which was ranked second with 89 publications. Italy, Germany, and India were also included in the top five. The high centrality score of the United States (0.35) indicates a high level of citations by other authors when compared to the other countries. However, this could be explained by the sheer number of publications by the United States or the filtering of English-only results. However, the language filter excluded less than 4% of our dataset. The analysis of publications by journal showed a greater number of studies coming from those that are neurosurgery focused. Out of the top 10 most cited, only two focused on craniofacial surgery, and one focused on plastic and reconstructive surgery.

The implications of a bibliometric analysis are strong when used to focus future research directions. The shift toward material review and clinical complications and outcomes leaves room of innovation in terms of procedure technique and postoperative care. Research into mitigating common complications on a material-dependent basis would allow for potential standardization of material type. In terms of authors, the limited direct collaboration between authors suggests opportunities for more interdisciplinary research. Future studies could focus on integrating expertise from different fields, such as materials science and surgical techniques, to advance cranioplasty. Similarly, with recent authors making significant impacts, more comprehensive reviews could be conducted in the next few years to consolidate the existing literature. Further, research combining results from various countries and regions could yield insightful results. The low levels of existing international contribution may be limiting the relevance of clinical outcome studies, as generalizations cannot be made without a large scale analysis. Future research could aim to conglomerate clinical outcome data and examine similarities and differences across regions.

Ultimately, the results of this bibliometric analysis reveal a clear shift toward clinical application and results of CP and away from methodology. This paper serves as a benchmark for prospective researchers to formulate new research ideas to further clinical understandings of how CP can be manipulated for better outcomes and less complications. By explicitly linking bibliometric trends to surgical practice, this study demonstrates that evolving literature can shape material selection and operative planning with the ultimate goal of optimizing patient outcomes.

## Limitations

The limitations of this analysis reside in its scope. We only used one database due to software limitations, and Scopus, while highly comprehensive, may not include articles in other popular databases such as World of Science and PubMed. However, Scopus is considered the most comprehensive database and remains the world’s largest [14, 38]. Similarly, our search query may have left out relevant articles that did not use the required keywords in their abstracts. However, our procedure falls within accepted standards and these limitations are common among bibliometric studies.

## Conclusion

Our CiteSpace-visualized bibliometric analysis shows that CP centered studies are currently on the rise with a focus on clinical outcomes and complication risk factors. Patient specific implants using alloplastic materials have dominated recent discourse and could soon be the predominant options over more traditional bone grafts and titanium mesh molds. Future focus will likely continue to investigate the positives and negatives of the various material options, and foster stronger collaboration between authors and countries, filling our identified research gaps. Based on overall trends, the next iteration of CP research is expected to examine optimization of patient outcomes in relation to material usage, and promote specific CP protocols to help standardize the extreme variety of CP application and material choice. Importantly, these evolving research directions matter because material selection and protocol standardization are directly tied to complication reduction, neurologic recovery, and long-term functional outcomes for patients [39].

## Supplementary Information

Below is the link to the electronic supplementary material.

Supplementary Material 1

## Data Availability

Data is provided within the manuscript and supplementary information files.
