# Supplementary material for: Cranial reconstruction in the literature: A CiteSpace visualized bibliometric analysis
Source: Neurosurg Rev. 2025 Oct 11;48(1):695. doi: 10.1007/s10143-025-03878-3 (PMC12515202; doi:10.1007/s10143-025-03878-3)
Supplement: Supplementary file 1 — Supplementary Material 1 [file 10143_2025_3878_MOESM1_ESM.docx]

**Supplementary Data**

Search Query: ( TITLE-ABS-KEY ( "craniofacial reconstruction" ) OR TITLE-ABS-KEY ( "cranioplasty" ) OR TITLE-ABS-KEY ( "cranial reconstruction" ) OR TITLE-ABS-KEY ( "skull reconstruction" ) OR TITLE-ABS-KEY ( "calvarial reconstruction" ) AND TITLE-ABS-KEY ( "alloplastic material" ) OR TITLE-ABS-KEY ( "titanium" ) OR TITLE-ABS-KEY ( "titanium mesh" ) OR TITLE-ABS-KEY ( "polymethyl methacrylate" ) OR TITLE-ABS-KEY ( "porous polyethylene" ) OR TITLE-ABS-KEY ( "bone cement" ) OR TITLE-ABS-KEY ( "autologous bone" ) OR TITLE-ABS-KEY ( "vascularized bone" ) OR TITLE-ABS-KEY ( "non-vascularized bone" ) OR TITLE-ABS-KEY ( "avascular bone" ) OR TITLE-ABS-KEY ( "bone graft" ) OR TITLE-ABS-KEY ( "polyetheretherketone" ) OR TITLE-ABS-KEY ( "PEEK" ) OR TITLE-ABS-KEY ( "MMA" ) OR TITLE-ABS-KEY ( "hydroxyapatite" ) ) AND PUBYEAR > 2013 AND PUBYEAR < 2025 AND ( LIMIT-TO ( DOCTYPE , "ar" ) OR LIMIT-TO ( DOCTYPE , "re" ) ) AND ( LIMIT-TO ( EXACTKEYWORD , "Human" ) OR LIMIT-TO ( EXACTKEYWORD , "Humans" ) ) AND ( LIMIT-TO ( LANGUAGE , "English" ) ).

**Supplementary Figure 1**

Top keyword frequency visualization.


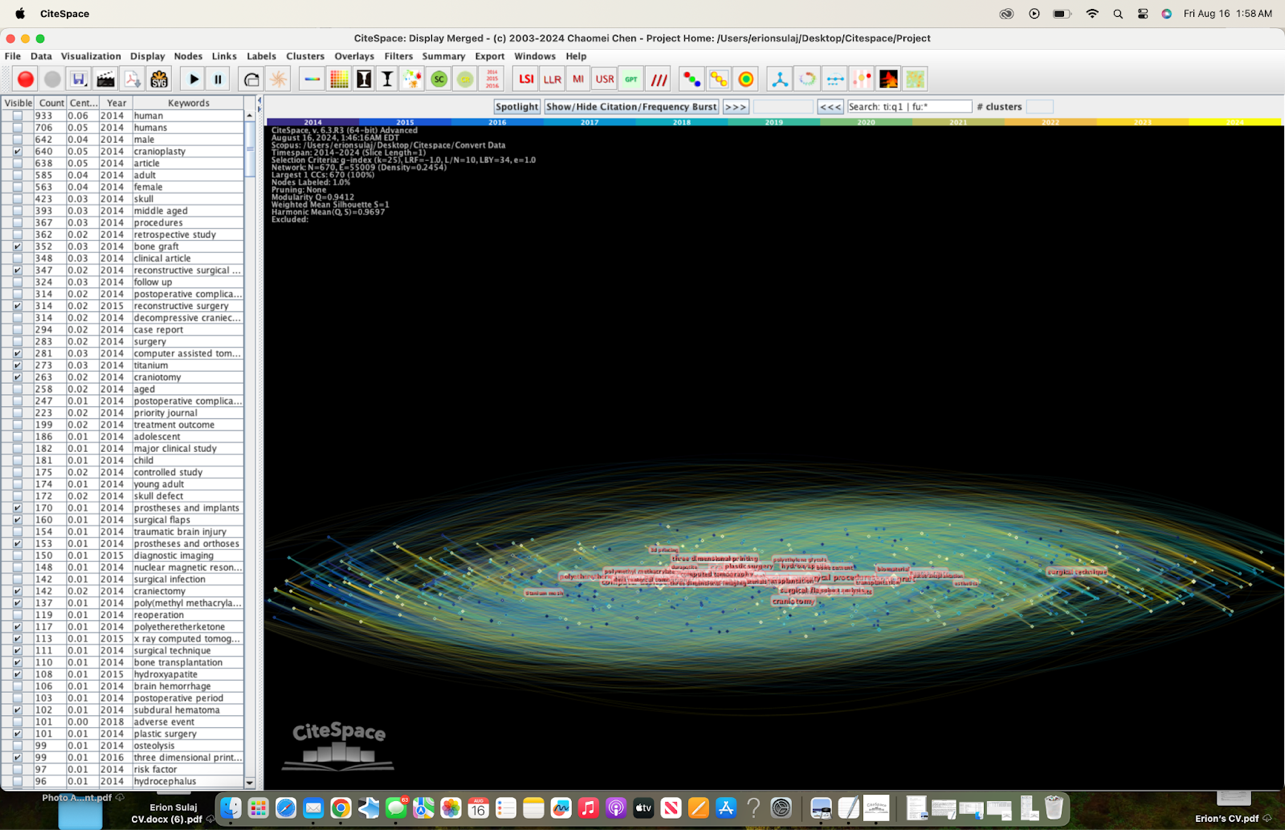


**Supplementary Table 1**

Full Keyword Summary Table

| **Rank** | **Keyword** | **Frequent** | **Centrality** |
| --- | --- | --- | --- |
| 1 | human | 933 | 0.06 |
| 2 | humans | 706 | 0.05 |
| 3 | male | 642 | 0.04 |
| 4 | cranioplasty | 640 | 0.05 |
| 5 | article | 638 | 0.05 |
| 6 | adult | 585 | 0.04 |
| 7 | female | 563 | 0.04 |
| 8 | skull | 423 | 0.03 |
| 9 | middle aged | 393 | 0.03 |
| 10 | procedures | 367 | 0.03 |
| 11 | retrospective study | 362 | 0.02 |
| 12 | bone graft | 352 | 0.03 |
| 13 | clinical article | 348 | 0.03 |
| 14 | reconstructive surgical procedures | 347 | 0.02 |
| 15 | follow up | 324 | 0.03 |
| 16 | decompressive craniectomy | 314 | 0.02 |
| 17 | postoperative complication | 314 | 0.02 |
| 18 | reconstructive surgery | 314 | 0.02 |
| 19 | case report | 294 | 0.02 |
| 20 | surgery | 283 | 0.02 |
| 21 | computer assisted tomography | 281 | 0.03 |
| 22 | titanium | 273 | 0.03 |
| 23 | craniotomy | 263 | 0.02 |
| 24 | aged | 258 | 0.02 |
| 25 | postoperative complications | 247 | 0.01 |
| 26 | priority journal | 223 | 0.02 |
| 27 | treatment outcome | 199 | 0.02 |
| 28 | adolescent | 186 | 0.01 |
| 29 | major clinical study | 182 | 0.01 |
| 30 | child | 181 | 0.01 |
| 31 | controlled study | 175 | 0.02 |
| 32 | young adult | 174 | 0.01 |
| 33 | skull defect | 172 | 0.02 |
| 34 | prostheses and implants | 170 | 0.01 |
| 35 | surgical flaps | 160 | 0.01 |
| 36 | traumatic brain injury | 154 | 0.01 |
| 37 | prostheses and orthoses | 153 | 0.01 |
| 38 | diagnostic imaging | 150 | 0.01 |
| 39 | nuclear magnetic resonance imaging | 148 | 0.01 |
| 40 | craniectomy | 142 | 0.02 |
| 41 | surgical infection | 142 | 0.01 |
| 42 | poly(methyl methacrylate) | 137 | 0.01 |
| 43 | reoperation | 119 | 0.01 |
| 44 | polyetheretherketone | 117 | 0.01 |
| 45 | x ray computed tomography | 113 | 0.01 |
| 46 | surgical technqiue | 111 | 0.01 |
| 47 | bone transplantation | 110 | 0.01 |
| 48 | hydroxyapatite | 108 | 0.01 |
| 49 | brain hemorrhage | 106 | 0.01 |
| 50 | postoperative period | 103 | 0.01 |
| 51 | subdural hematoma | 102 | 0.01 |
| 52 | plastic surgery | 101 | 0.01 |
| 53 | adverse event | 101 | 0 |
| 54 | osteolysis | 99 | 0.01 |
| 55 | three dimensional printing | 99 | 0.01 |
| 56 | risk factor | 97 | 0.01 |
| 57 | hydrocephalus | 96 | 0.01 |
| 58 | preschool child | 92 | 0.01 |
| 59 | surgical mesh | 88 | 0.01 |
| 60 | cohort analysis | 86 | 0.01 |
| 61 | liquorrhea | 86 | 0.01 |
| 62 | operative duration | 83 | 0.01 |
| 63 | review | 83 | 0.01 |
| 64 | postoperative infection | 80 | 0.01 |
| 65 | clinical outcome | 80 | 0.01 |
| 66 | antibiotic agent | 79 | 0.01 |
| 67 | biomaterial | 79 | 0.01 |
| 68 | polymethyl methacrylate | 79 | 0.01 |
| 69 | neurosurgery | 78 | 0.01 |
| 70 | outcome assessment | 77 | 0.01 |
| 71 | seizure | 77 | 0.01 |
| 72 | human tissue | 77 | 0.01 |
| 73 | tomography | 75 | 0 |
| 74 | bone cement | 73 | 0.01 |
| 75 | epidural hematoma | 73 | 0.01 |
| 76 | dura mater | 71 | 0.01 |
| 77 | glasgow coma scale | 71 | 0 |
| 78 | hematoma | 70 | 0 |
| 79 | biocompatible materials | 69 | 0 |
| 80 | brain ventricle peritoneum shunt | 69 | 0 |
| 81 | pathology | 67 | 0 |
| 82 | transplantation | 66 | 0 |
| 83 | follow up study | 64 | 0 |
| 84 | surgical wound infection | 64 | 0 |
| 85 | headache | 63 | 0.01 |
| 86 | comparative study | 63 | 0 |
| 87 | meningioma | 61 | 0.01 |
| 88 | three dimensional imaging | 60 | 0.01 |
| 89 | infant | 60 | 0 |
| 90 | complication | 59 | 0 |
| 91 | subarachnoid hemorrhage | 59 | 0 |
| 92 | devices | 58 | 0 |
| 93 | very elderly | 58 | 0 |
| 94 | brain edema | 55 | 0 |
| 95 | cancer surgery | 55 | 0.01 |
| 96 | plastic surgery procedures | 55 | 0 |
| 97 | head injury | 54 | 0 |
| 98 | patient satisfaction | 53 | 0 |
| 99 | scalp | 53 | 0 |
| 100 | ketones | 52 | 0 |
| 101 | prospective study | 52 | 0 |
| 102 | antibiotic therapy | 51 | 0 |
| 103 | computer aided design | 51 | 0 |
| 104 | ketones | 50 | 0 |
| 105 | risk factors | 50 | 0 |
| 106 | neuroimaging | 49 | 0 |
| 107 | printing | 49 | 0 |
| 108 | adverse effects | 48 | 0 |
| 109 | bone resorption | 48 | 0 |
| 110 | brain tumor | 48 | 0 |
| 111 | frontal bone | 48 | 0 |
| 112 | esthetics | 47 | 0 |
| 113 | polyethylene glycols | 47 | 0 |
| 114 | bone defect | 46 | 0 |
| 115 | autotransplantation | 45 | 0 |
| 116 | 3d printing | 44 | 0 |
| 117 | durapatite | 43 | 0 |
| 118 | brain injuries | 43 | 0 |
| 119 | histopathology | 42 | 0 |
| 120 | intracranial pressure | 42 | 0 |
| 121 | length of stay | 42 | 0 |
| 122 | titanium mesh | 42 | 0 |

**Supplementary Table 2**

Country citation frequency and centrality

| **Ranking** | **Country** | **Citations** | **Centrality** |
| --- | --- | --- | --- |
| 1 | United States | 256 | 0.35 |
| 2 | China | 89 | 0.04 |
| 3 | Italy | 71 | 0.23 |
| 4 | Germany | 65 | 0.21 |
| 5 | India | 61 | 0.02 |
| 6 | Japan | 53 | 0 |
| 7 | South Korea | 44 | 0 |
| 8 | United Kingdom | 41 | 0.17 |
| 9 | France | 34 | 0 |
| 10 | Netherlands | 24 | 0.02 |
